# Survival analysis across the entire transcriptome identifies biomarkers with the highest prognostic power in breast cancer

**DOI:** 10.1016/j.csbj.2021.07.014

**Published:** 2021-07-18

**Authors:** Balázs Győrffy

**Affiliations:** a Semmelweis University Dept. of Bioinformatics, Tűzoltó utca 7-9., 1094 Budapest, Hungary; b TTK Momentum Cancer Biomarker Research Group, Institute of Enzymology, Magyar Tudósok körútja 2., 1117 Budapest, Hungary; c Semmelweis University 2nd Dept. of Pediatrics, Tűzoltó utca 7-9., 1094 Budapest, Hungary

**Keywords:** Survival, Breast cancer, Chemotherapy, Biomarkers, Prognosis, Kaplan-Meier plot, Molecular subtype

## Abstract

**Introduction:**

Extensive research is directed to uncover new biomarkers capable to stratify breast cancer patients into clinically relevant cohorts. However, the overall performance ranking of such marker candidates compared to other genes is virtually absent. Here, we present the ranking of all survival related genes in chemotherapy treated basal and estrogen positive/HER2 negative breast cancer.

**Methods:**

We searched the GEO repository to uncover transcriptomic datasets with available follow-up and clinical data. After quality control and normalization, samples entered an integrated database. Molecular subtypes were designated using gene expression data. Relapse-free survival analysis was performed using Cox proportional hazards regression. False discovery rate was computed to combat multiple hypothesis testing. Kaplan-Meier plots were drawn to visualize the best performing genes.

**Results:**

The entire database includes 7,830 unique samples from 55 independent datasets. Of those with available relapse-free survival time, 3,382 samples were estrogen receptor-positive and 696 were basal. In chemotherapy treated ER positive/ERBB2 negative patients the significant prognostic biomarker genes achieved hazard rates between 1.76 and 3.33 with a p value below 5.8E−04. The significant prognostic genes in adjuvant chemotherapy treated basal breast cancer samples reached hazard rates between 1.88 and 3.61 with a p value below 7.2E−04. Our integrated platform was extended enabling the validation of future biomarker candidates.

**Conclusions:**

A reference ranking for all genes in two chemotherapy treated breast cancer cohorts is presented. The results help to neglect those with unlikely clinical significance and to focus future research on the most promising candidates.

## Graphical abstract

**Figure:**
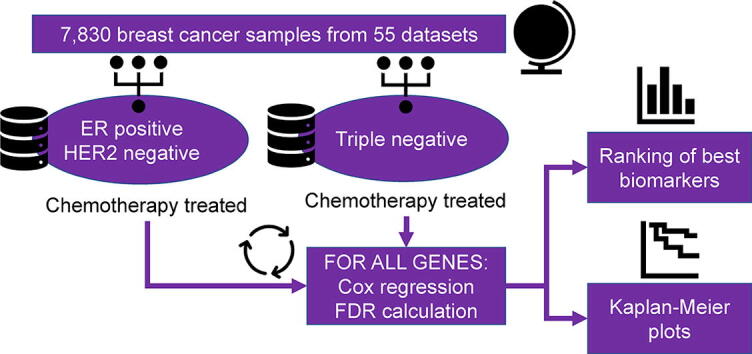

## 1 Introduction

Breast cancer is by far the most common cancer in women [1] with two main established molecular biomarkers for systemic therapy, estrogen receptor (ER) alpha and epidermal growth receptor 2 (ERBB2/HER2). These define three distinct molecular subtypes, the ER alpha positive/ERBB2 negative, the ERBB2 positive, and the basal tumors (these lack ER alpha, ERBB2, and also progesterone receptor). Progesterone receptor (PR) is a gene regulated by ER alpha and ER alpha positive/PR negative cancers are exceedingly rare [2].

ER positive tumors are treated with hormone therapy and occasionally with some chemotherapy, ERBB2 positive tumors are treated with anti-ERBB2 therapy and chemotherapy and basal tumors receive chemotherapy only [3]. With the introduction of anti-ERBB2 therapies the previously inferior prognosis of ERBB2 positive patients improved dramatically [4]. Today, basal cases have the worst expected outcome with high risk of relapse within a few years following diagnosis [5]. The ER positive/ERBB2 negative patients represent 70% of all cases, the ERBB2 positive cases account for 15–20%, and the basal cases denote 15% of all breast cancer cases [6].

In recent years few new agents have been approved, including CDK4/6 inhibitors for the treatment of advanced ER positive patients [7] and PARP inhibitors for patients with germline BRCA mutation [8]. However, despite multiple large-scale tumor sequencing studies, germline mutations in BRCA1 and BRCA2 [9] remain the solitary mutations capable to serve as basis for clinically valuable targeted therapy. At the same time, monogenic gene expression based predictive biomarkers have been supplemented by new generations of multigenic prognostic test. Some of the multigenic tests claim to predict both early and late relapse [10].

The most important genes used today as predictive markers (capable to serve as biomarkers predicting response to a given agent) emerged first as prognostic biomarkers (genes capable to predict the expected survival of the patients). This was the case for both ER [11] and ERBB2 [12]. Considering the efficiency of current systemic therapies, the next level should lie in investigation of patient cohorts further stratified based on administered treatment – which itself is already based on the currently approved clinical markers. To achieve this goal, one has to identify and rank all prognostic biomarkers. Here, we aimed to perform such an analysis using publicly available gene expression datasets in basal and in the estrogen-positive/ERBB2 negative chemotherapy treated breast cancer.

## 2 Methods

### 2.1 Database setup

We performed a search in the GEO (https://www.ncbi.nlm.nih.gov/geo/) and EGA (https://ega-archive.org/) repositories to identify transcriptome-level gene expression datasets with available clinical information. In this, only datasets with at least 30 samples were considered and only those which were generated using the GEO platforms GPL96, GPL570, and GPL571. The reason for this filter is that these platforms have an overlapping set of 22,277 genes measured using the exact same probe sequences. It is only possible to have the same sensitivity, specificity, and dynamic range in case the same probe sets are used.

### 2.2 Quality control and pre-processing

First, each array was normalized using MAS5 – we selected MAS5 as it ranked among the best performing normalization techniques in our previous comparison of available methods using RT-PCR validated expression values [13]. In addition, MAS5 enables the normalization of a single sample separately, thus the insertion or removal of a sample or samples does not affect the other values within the dataset. Then, a second scaling normalization was performed to reduce batch effects by setting mean expression of the overlapping 22,277 probes to 1000 in each array [14].

In order to remove redundant samples, the normalized gene expression values across all samples were compared. In case of identical expression values, only the first publication of a given gene array was retained in the database, and all subsequent copies were removed. Five parameters were analyzed for quality control: the background, the raw Q, the percentage of present calls, the presence of bioBCD spikes, and the GAPDH/ACTB 3 to 5 ratio. Samples with positive values and – for continuous variables – those within the 95% range for all samples passed the quality control. Those where one parameter did not pass, were designated as outliers, and those where two or more parameters did not pass were marked as biased arrays. Biased arrays were excluded from the subsequent statistical analyses.

### 2.3 Molecular subtype determination

Molecular subtypes were determined using the StGallen criteria [15]. Because only the gene expression measured on the gene arrays were available for all samples, these were used to determine receptor status for each patient. In this, the cutoff of 500 for the probe set 205225_at was used to determine estrogen positivity and the probe set 216836_s_at with a cutoff of 4800 was used to assign patients into ERBB2 positive/negative groups [16]. Progesterone receptor was not included, because there is no reliable probe set for this gene in the GPL96 gene arrays. Present analysis was restricted to two systemically treated cohorts, to those who are estrogen positive ERBB2 negative and to those who are negative for both estrogen and ERBB2 receptors.

### 2.4 Survival analysis

Cox proportional hazards regression analysis was made for each gene separately. In this, each possible cutoff value was examined between the lower and upper quartiles, and False-Discovery Rate using the Benjamini-Hochberg method was computed to correct for multiple hypothesis testing. The survival analysis was performed for relapse-free survival (RFS). Breast cancer specific survival was not used because almost all studies published OS and/or RFS only. In case of identical p values the strongest hazard rate was identified. The results for the best performing cutoff were exported for each gene in a separate database, and these were used to generate Kaplan-Meier plots to visualize correlation between gene expression and survival.

### 2.5 Gene ontology analysis

We performed gene ontology analysis for the derived lists in each setting separately. In this, only the significant genes were included and The Database for Annotation, Visualization and Integrated Discovery (DAVID) tool was used to uncover over-represented biological processes (BP) and molecular functions (MF) [17]. Only hits with a Benjamini-Hochberg False Discovery Rate below 0.05 were accepted as significant.

### 2.6 Updates of www.kmplot.com

Our database was initially established in 2010 with 1809 patients and at that time we also established an online survival analysis platform to enable the investigation of the assembled dataset by independent researchers [18]. In addition to updating the database in the online analysis platform new analysis options were also added to the site, including the cutoff determination algorithm and molecular subtypes utilized in present manuscript.

## 3 Results

### 3.1 Database

**Fig. 1:**
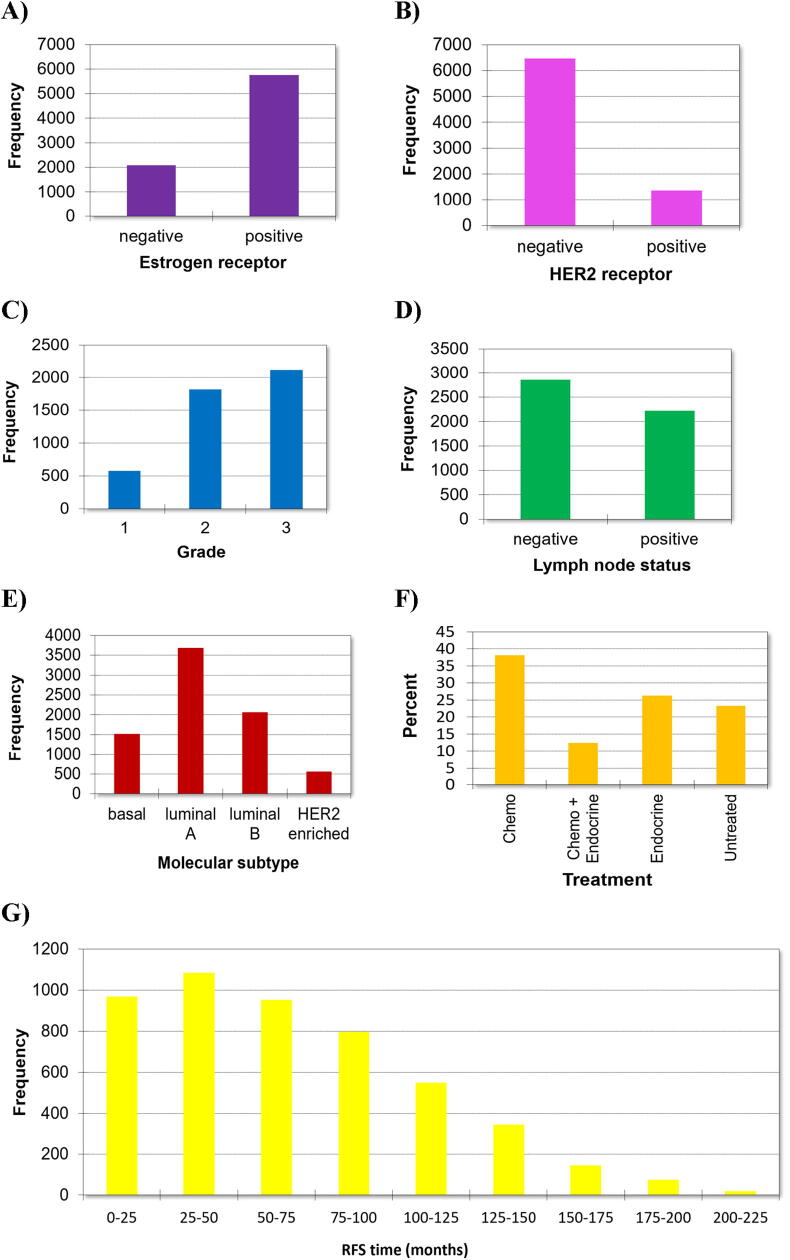
Descriptive characteristics of the entire database including distribution of estrogen receptor status (A), HER2 receptor status (B), grade (C), nodal involvement (D), molecular subtypes (E), treatment (F), and follow-up for relapse-free survival (G).

The total number of breast cancer arrays was 9423 and these represent 7830 unique samples from 55 independent datasets. Of these, there were 1139 outliers and 77 biased arrays. All biased arrays were excluded from further analysis. Relapse-free survival was available for 5268 patients and overall survival time for 5165 patients. Clinical characteristics for the entire database are presented in Table 1. Of note, the total sample number in Table 1 for some studies is lower due to the exclusion of redundant samples, as described in the Methods section. Clinical characteristics of the entire database including receptor status, grade, lymph node status, molecular subtype distribution, applied treatment and length of follow-up for relapse-free survival are summarized in Fig. 1.

**Table 1:** An overview of the clinical characteristics of all datasets integrated into the complete database. *NA: no data, RFS: relapse-free survival, OS: overall survival, ER: estrogen receptor, MTAB-365: E-MTAB-365 dataset, TABM-43: E-TABM-43 dataset.*

Dataset	Sample		RFS		OS		ER +		ERBB2 +		Node negative		Basal		Luminal A		Luminal B		ERBB2		Grade 1		Grade 2		Grade 3	
	n	%	n	months	n	months	n	%	n	%	n	%	n	%	n	%	n	%	n	%	n	%	n	%	n	%
**GSE11121**	200	2.6%	200	93.9 ± 7.1	NA	NA	181	90.5%	25	12.5%	200	100.0%	15	7.5%	130	65.0%	51	25.5%	4	2.0%	29	14.5%	136	68.0%	35	17.5%
**GSE12093**	136	1.7%	136	92.3 ± 6.5	NA	NA	136	100.0%	14	10.3%	136	100.0%	0	0.0%	104	76.5%	32	23.5%	0	0.0%	0	NA	0	NA	0	NA
**GSE12276**	204	2.6%	204	26.2 ± 3.0	NA	NA	127	62.3%	48	23.5%	0	0.0%	57	27.9%	70	34.3%	57	27.9%	20	9.8%	0	NA	0	NA	0	NA
**GSE1456**	159	2.0%	159	74.4 ± 4.3	159	76.8 ± 3.6	141	88.7%	23	14.5%	0	0.0%	17	10.7%	43	27.0%	98	61.6%	1	0.6%	28	19.0%	58	39.5%	61	41.5%
**GSE16391**	55	0.7%	48	34.7 ± 4.3	NA	NA	54	98.2%	3	5.5%	22	40.0%	1	1.8%	46	83.6%	8	14.5%	0	0.0%	2	3.6%	35	63.6%	18	32.7%
**GSE16446**	120	1.5%	107	35.7 ± 3.5	107	38.6 ± 3.2	9	7.5%	28	23.3%	55	45.8%	86	71.7%	5	4.2%	4	3.3%	25	20.8%	2	1.7%	20	16.7%	92	76.7%
**GSE16716**	47	0.6%	8	61.1 ± 25.6	7	68.4 ± 22.8	30	63.8%	32	68.1%	2	25.0%	4	8.5%	6	12.8%	24	51.1%	13	27.7%	0	0.0%	13	27.7%	34	72.3%
**GSE17705**	196	2.5%	196	105.6 ± 6.1	NA	NA	191	97.4%	10	5.1%	111	56.6%	5	2.6%	98	50.0%	93	47.4%	0	0.0%	0	NA	0	NA	0	NA
**GSE17907**	54	0.7%	38	39.7 ± 9.6	NA	NA	28	51.9%	48	88.9%	14	31.8%	0	0.0%	6	11.1%	22	40.7%	26	48.1%	3	6.5%	9	19.6%	34	73.9%
**GSE18728**	61	0.8%	NA	NA	NA	NA	45	73.8%	6	9.8%	0	NA	14	23.0%	35	57.4%	10	16.4%	2	3.3%	0	NA	0	NA	0	NA
**GSE19615**	115	1.5%	115	60.0 ± 4.0	NA	NA	75	65.2%	26	22.6%	62	53.9%	31	27.0%	47	40.9%	28	24.3%	9	7.8%	23	20.0%	28	24.3%	64	55.7%
**GSE20194**	45	0.6%	NA	NA	NA	NA	29	64.4%	16	35.6%	9	25.0%	9	20.0%	10	22.2%	19	42.2%	7	15.6%	0	0.0%	8	23.5%	26	76.5%
**GSE20271**	96	1.2%	2	21.4 ± 13.6	2	21.4 ± 13.6	66	68.8%	15	15.6%	38	39.6%	21	21.9%	18	18.8%	48	50.0%	9	9.4%	5	6.6%	30	39.5%	41	53.9%
**GSE2034**	286	3.7%	286	77.5 ± 4.9	NA	NA	229	80.1%	61	21.3%	286	100.0%	44	15.4%	131	45.8%	98	34.3%	13	4.5%	0	NA	0	NA	0	NA
**GSE20685**	327	4.2%	327	87.6 ± 4.7	327	94.7 ± 4.2	261	79.8%	89	27.2%	0	NA	37	11.3%	165	50.5%	96	29.4%	29	8.9%	0	NA	0	NA	0	NA
**GSE20711**	90	1.1%	88	67.4 ± 9.1	88	83.0 ± 7.6	58	64.4%	21	23.3%	29	32.2%	19	21.1%	47	52.2%	11	12.2%	13	14.4%	13	14.4%	5	5.6%	70	77.8%
**GSE21653**	240	3.1%	230	60.8 ± 5.5	NA	NA	158	65.8%	29	12.1%	111	46.4%	77	32.1%	93	38.8%	65	27.1%	5	2.1%	44	18.3%	82	34.2%	108	45.0%
**GSE22093**	68	0.9%	NA	NA	31	58.8 ± 10.9	39	57.4%	17	25.0%	18	26.5%	21	30.9%	8	11.8%	31	45.6%	8	11.8%	2	2.9%	19	27.9%	39	57.4%
**GSE23988**	8	0.1%	NA	NA	NA	NA	6	75.0%	0	0.0%	1	12.5%	2	25.0%	2	25.0%	4	50.0%	0	0.0%	0	0.0%	3	37.5%	4	50.0%
**GSE25066**	507	6.5%	507	35.8 ± 1.7	NA	NA	360	71.0%	10	2.0%	169	33.8%	142	28.0%	135	26.6%	225	44.4%	5	1.0%	32	6.5%	179	36.5%	259	52.7%
**GSE2603**	99	1.3%	82	62.1 ± 6.2	NA	NA	66	66.7%	15	15.2%	34	34.3%	29	29.3%	18	18.2%	48	48.5%	4	4.0%	0	NA	0	NA	0	NA
**GSE26971**	276	3.5%	97	71.0 ± 6.9	NA	NA	270	97.8%	9	3.3%	131	47.5%	5	1.8%	224	81.2%	46	16.7%	1	0.4%	12	12.9%	62	66.7%	19	20.4%
**GSE29044**	79	1.0%	NA	NA	NA	NA	63	79.7%	17	21.5%	0	NA	5	6.3%	53	67.1%	10	12.7%	11	13.9%	3	8.3%	18	50.0%	15	41.7%
**GSE2990**	102	1.3%	102	84.1 ± 10.3	NA	NA	89	87.3%	17	16.7%	85	83.3%	7	6.9%	56	54.9%	33	32.4%	6	5.9%	27	32.5%	20	24.1%	36	43.4%
**GSE31448**	71	0.9%	NA	NA	NA	NA	39	54.9%	3	4.2%	17	65.4%	30	42.3%	19	26.8%	20	28.2%	2	2.8%	0	NA	0	NA	0	NA
**GSE31519**	67	0.9%	64	39.6 ± 6.5	NA	NA	19	28.4%	5	7.5%	44	67.7%	45	67.2%	5	7.5%	14	20.9%	3	4.5%	18	28.6%	0	0.0%	45	71.4%
**GSE32646**	115	1.5%	NA	NA	NA	NA	79	68.7%	23	20.0%	32	27.8%	23	20.0%	49	42.6%	30	26.1%	13	11.3%	16	13.9%	78	67.8%	21	18.3%
**GSE3494**	251	3.2%	249	85.1 ± 6.4	236	98.0 ± 5.9	228	90.8%	45	17.9%	158	62.9%	17	6.8%	138	55.0%	90	35.9%	6	2.4%	67	26.9%	128	51.4%	54	21.7%
**GSE36771**	107	1.4%	NA	NA	NA	NA	79	73.8%	15	14.0%	45	42.1%	19	17.8%	66	61.7%	13	12.1%	9	8.4%	11	10.3%	42	39.3%	54	50.5%
**GSE37946**	41	0.5%	40	54.0 ± 6.0	40	54.0 ± 6.0	27	65.9%	32	78.0%	33	80.5%	5	12.2%	2	4.9%	25	61.0%	9	22.0%	0	0.0%	10	25.0%	30	75.0%
**GSE41998**	279	3.6%	NA	NA	NA	NA	141	50.5%	24	8.6%	0	NA	118	42.3%	126	45.2%	15	5.4%	20	7.2%	0	NA	0	NA	0	NA
**GSE42568**	121	1.5%	104	54.4 ± 6.3	104	63.3 ± 5.7	91	75.2%	15	12.4%	45	37.2%	23	19.0%	73	60.3%	18	14.9%	7	5.8%	11	9.1%	40	33.1%	53	43.8%
**GSE42822**	91	1.2%	NA	NA	NA	NA	54	59.3%	30	33.0%	29	33.0%	20	22.0%	41	45.1%	13	14.3%	17	18.7%	0	0.0%	23	30.3%	53	69.7%
**GSE43358**	57	0.7%	NA	NA	NA	NA	38	66.7%	9	15.8%	0	NA	16	28.1%	31	54.4%	7	12.3%	3	5.3%	16	28.1%	6	10.5%	35	61.4%
**GSE43365**	111	1.4%	NA	NA	NA	NA	95	85.6%	13	11.7%	85	76.6%	10	9.0%	84	75.7%	11	9.9%	6	5.4%	21	18.9%	54	48.6%	36	32.4%
**GSE45255**	139	1.8%	94	49.9 ± 5.0	134	54.9 ± 3.6	118	84.9%	31	22.3%	94	67.6%	13	9.4%	47	33.8%	71	51.1%	8	5.8%	17	12.2%	52	37.4%	67	48.2%
**GSE4611**	153	2.0%	152	43.8 ± 3.0	NA	NA	133	86.9%	40	26.1%	79	52.3%	2	1.3%	81	52.9%	52	34.0%	18	11.8%	16	10.5%	89	58.6%	47	30.9%
**GSE46184**	74	0.9%	74	72.4 ± 7.8	NA	NA	60	81.1%	32	43.2%	42	56.8%	2	2.7%	23	31.1%	37	50.0%	12	16.2%	1	1.4%	35	47.9%	37	50.7%
**GSE48390**	81	1.0%	81	44.8 ± 3.7	81	44.8 ± 3.7	64	79.0%	13	16.0%	0	NA	9	11.1%	52	64.2%	12	14.8%	8	9.9%	0	NA	0	NA	0	NA
**GSE4922**	1	0.0%	1	146.0	NA	NA	1	100.0%	0	0.0%	1	100.0%	0	0.0%	1	100.0%	0	0.0%	0	0.0%	1	100.0%	0	0.0%	0	0.0%
**GSE50948**	156	2.0%	NA	NA	NA	NA	83	53.2%	77	49.4%	0	NA	28	17.9%	37	23.7%	46	29.5%	45	28.8%	0	0.0%	67	43.8%	86	56.2%
**GSE5327**	58	0.7%	58	81.3 ± 9.7	NA	NA	22	37.9%	12	20.7%	0	0.0%	30	51.7%	3	5.2%	19	32.8%	6	10.3%	0	NA	0	NA	0	NA
**GSE5462**	116	1.5%	NA	NA	NA	NA	116	100.0%	3	2.6%	0	NA	0	0.0%	113	97.4%	3	2.6%	0	0.0%	0	NA	0	NA	0	NA
**GSE58812**	107	1.4%	NA	NA	107	73.4 ± 8.0	21	19.6%	3	2.8%	0	NA	83	77.6%	21	19.6%	0	0.0%	3	2.8%	0	NA	0	NA	0	NA
**GSE61304**	62	0.8%	58	30.3 ± 5.0	NA	NA	40	64.5%	16	25.8%	20	35.1%	15	24.2%	30	48.4%	10	16.1%	7	11.3%	5	8.6%	16	27.6%	37	63.8%
**GSE65194**	164	2.1%	130	49.0 ± 3.9	130	50.7 ± 3.8	83	50.6%	64	39.0%	58	52.3%	46	28.0%	44	26.8%	39	23.8%	35	21.3%	0	NA	0	NA	0	NA
**GSE6532**	82	1.0%	77	72.7 ± 8.4	NA	NA	80	97.6%	11	13.4%	52	63.4%	0	0.0%	63	76.8%	17	20.7%	2	2.4%	0	0.0%	54	98.2%	1	1.8%
**GSE66305**	88	1.1%	NA	NA	NA	NA	54	61.4%	59	67.0%	0	NA	6	6.8%	22	25.0%	32	36.4%	28	31.8%	0	NA	0	NA	0	NA
**GSE69031**	130	1.7%	129	68.4 ± 8.4	129	76.5 ± 7.7	98	75.4%	21	16.2%	59	45.4%	27	20.8%	76	58.5%	22	16.9%	5	3.8%	14	11.2%	46	36.8%	65	52.0%
**GSE7390**	198	2.5%	198	111.7 ± 9.3	198	136.4 ± 8.4	143	72.2%	29	14.6%	198	100.0%	40	20.2%	119	60.1%	24	12.1%	15	7.6%	30	15.3%	83	42.3%	83	42.3%
**GSE76275**	265	3.4%	NA	NA	NA	NA	140	52.8%	10	3.8%	74	49.3%	121	45.7%	101	38.1%	39	14.7%	4	1.5%	5	2.4%	80	37.7%	127	59.9%
**GSE78958**	424	5.4%	NA	NA	NA	NA	334	78.8%	45	10.6%	0	NA	71	16.7%	246	58.0%	88	20.8%	19	4.5%	88	20.9%	156	37.0%	178	42.2%
**GSE9195**	77	1.0%	77	93.2 ± 6.8	NA	NA	75	97.4%	7	9.1%	41	53.2%	1	1.3%	65	84.4%	10	13.0%	1	1.3%	14	18.2%	20	26.0%	24	31.2%
**MTAB-365**	537	6.9%	426	73.3 ± 4.8	429	79.2 ± 4.7	460	85.7%	89	16.6%	139	25.9%	45	8.4%	345	64.2%	115	21.4%	32	6.0%	0	NA	0	NA	0	NA
**TABM-43**	37	0.5%	NA	NA	NA	NA	24	64.9%	10	27.0%	0	NA	7	18.9%	14	37.8%	10	27.0%	6	16.2%	0	0.0%	14	37.8%	23	62.2%

	**Sample**		**RFS**		**OS**		**ER +**		**ERBB2 +**		**Node negative**		**Basal**		**Luminal A**		**Luminal B**		**ERBB2**		**Grade 1**		**Grade 2**		**Grade 3**	

**n**	**%**	**n**	**months**	**n**	**months**	**n**	**%**	**n**	**%**	**n**	**%**	**n**	**%**	**n**	**%**	**n**	**%**	**n**	**%**	**n**	**%**	**n**	**%**	**n**	**%**

**Combined**	**7830**	**100.0%**	**4944**	**67.3 ± 1.3**	**2309**	**80.2 ± 1.9**	**5750**	**73.4%**	**1365**	**17.4%**	**2858**	**36.5%**	**1520**	**19.4%**	**3687**	**47.1%**	**2063**	**26.3%**	**560**	**7.2%**	**576**	**12.8%**	**1818**	**40.4%**	**2111**	**46.9%**

In order to select the most reliable probes, multiple filtering steps were executed. First, only probe sets mapped to a gene were retained. Then a second filter was added to remove all genes with a false discovery rate over 5%. Then, a third and a fourth filter were set in which the maximal expression had to be over 1000 and the cutoff values had to be over 100, respectively. The goal of these filters was to include only genes which have robust expression suitable for independent validation. Finally, only the JetSet best probe sets [19] were retained.

Prognostic biomarkers in estrogen-positive, ERBB2 negative, chemotherapy treated breast tumors

Fifteen out of the 55 datasets had patient samples eligible for this analysis (these include the datasets GSE1456, GSE16391, GSE16446, GSE16716, GSE17907, GSE19615, GSE21653, GSE25066, GSE31519, GSE3494, GSE37946, GSE45255, GSE4611, GSE5327, and GSE69031). The cumulative number of patients included in these totaled at n = 712 (for some genes the n was 131 due to array platform differences). When running the Cox regression for relapse-free survival, there were 1496 genes below the 5% FDR threshold and 1257 of these had expression over 1000 in at least one sample. The threshold of 1000 was used as this was the mean expression for all genes after the normalization. The cutoff was over 100 for 1203 genes and 692 of these were JetSet best probe sets. The complete table of all significant genes ranked by absolute HR values is presented as Supplemental Table 1.

**Fig. 2:**
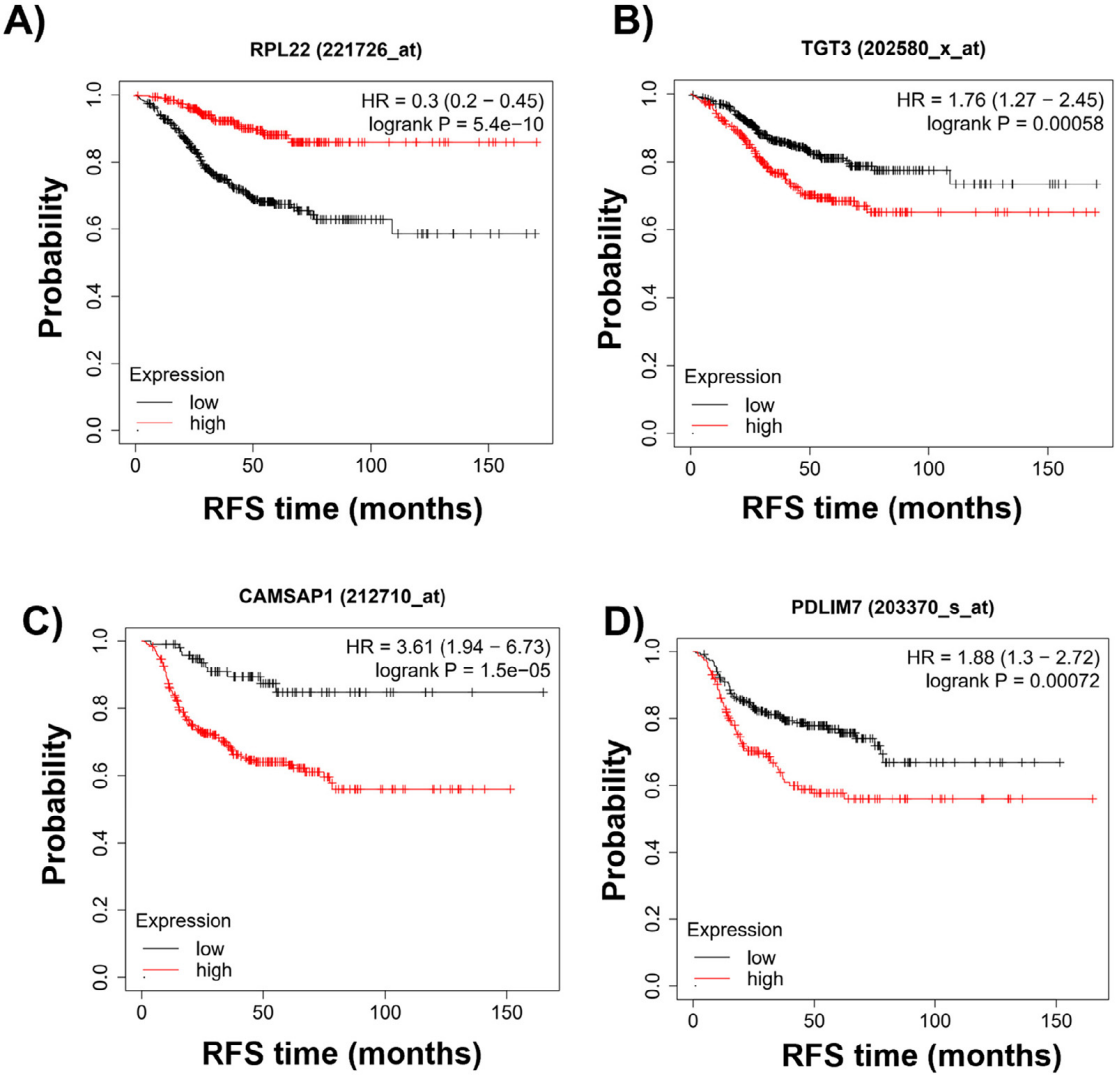
Genes related to relapse-free survival after chemotherapy. The *best performing* genes in chemotherapy treated ER positive HER2 negative breast cancer (A), and in all chemotherapy treated basal breast cancer (C). The *first genes reaching significance* after multiple testing correction in chemotherapy treated ER positive HER2 negative breast cancer (B) and in all chemotherapy treated basal tumors (D).

What is the maximal hazard rate a gene can achieve? We can estimate the potential effect of a gene when ranking all genes and selecting the most significant one. When investigating all genes in all patients in the estrogen receptor positive ERBB2 receptor negative cohort, Ribosomal Protein L22 (RPL22) reached the highest significance with a HR of 0.3 (higher expression of RPL22 was associated with better survival, and thus the value of 0.3 would equal to an absolute HR of 3.33) and a p of 5.4E−10 (Fig. 2A). The first significant gene was Thyroid transcription factor I (TGT3) with a HR of 1.76 and a p of 5.8E−04 (Fig. 2B). Genes with inferior p value did not reached statistical significance after multiple hypothesis testing (FDR over 5%).

Thirteen biological processes reached significance in the GO analysis, cell division (GO:0051301, p = 6.02E−11), mitotic sister chromatid segregation (GO:0000070, p = 5.21E−07), and cell proliferation (GO:0008283, p = 1.16E−06) reaching the lowest p values. Only three molecular functions were significant, including ATP binding (GO:0005524, p = 3.81E−06) and microtubule binding (GO:0008017, p = 7.51E−05).

### 3.2 Estrogen-positive ERBB2 negative breast cancer with untreated excluded

In this setting we included all estrogen positive and ERBB2 negative patients (n = 2823) and then excluded all samples with no information about treatment and also excluded all systemically untreated patients. Of note, the restriction was for systemic therapies only (chemotherapy and endocrine therapy) as there was no information available about radiation therapy. Twenty-three datasets had eligible patients (these include GSE12093, GSE12276, GSE1456, GSE16391, GSE16446, GSE16716, GSE17705, GSE17907, GSE19615, GSE21653, GSE25066, GSE26971, GSE2990, GSE31519, GSE3494, GSE37946, GSE45255, GSE4611, GSE46184, GSE5327, GSE6532, GSE69031, and GSE9195), and the final number of patients included was 1679. Of note, some genes were only present in the HGU133plus2 arrays, and therefore only patients who were measured by this platform were included (n = 384). Of the 37,535 probe sets mapping to a gene, 17,088 genes reached statistical significance at FDR < 5%. Of these, 11,029 had expression over 1000 in at least one sample, and the cutoff was over 100 for 8607 genes. When mapping to JetSet best probe sets, 4709 genes remained as significant, the complete list of these ranked by absolute HR values is provided in Supplemental Table 2.

Twenty biological processes reached significant over-representation among these genes including cell division (GO:0051301, p = 2.34E−11), proteasome-mediated ubiquitin-dependent protein catabolic process (GO:0043161, p = 5.92E−07), and regulation of signal transduction by p53 class mediator (GO:1901796, p = 7.63E−07). Poly(A)RNA binding (GO:0044822, p = 1.31E−53) and ATP binding (GO:0005524, p = 3.65E−07) were on the list of most important molecular functions. The complete lists of all the biological processes and molecular functions significant in each cohort are presented in Supplemental Table 3.

### 3.3 Genes associated with survival after chemotherapy in basal breast cancer

All together 13 datasets included basal breast cancers with documented chemotherapy, these include GSE1456, GSE16446, GSE16716, GSE19615, GSE21653, GSE25066, GSE31519, GSE3494, GSE37946, GSE45255, GSE4611, GSE5327, and GSE69031. The number of patient samples was 392. When running the survival analysis across all genes using relapse as the endpoint, only probe sets mapping to a gene were included (n = 37,535). Then filtering was made to include only those results where the False Discovery Rate was not higher than 5% (n = 652 genes remaining), and only those where the expression of the gene reached 1000 in at least one sample (n = 402 remaining). The cutoff designating high- and low-expression cohorts had to be over 100 (n = 380 remaining) to exclude probes with expression levels close to the background noise. Finally, the significant probe sets were reduced to include only the JetSet best probe sets (n = 246 remaining). The complete list of all results ranked by the absolute HR values is presented in Supplemental Table 4. In the gene ontology analysis extracellular matrix organization (GO:0030198, p = 1.33E−08) reached the highest significance.

When ranking all genes derived using all patients in this cohort, the most significant gene was Calmodulin-regulated spectrin-associated protein 1 (CAMSAP1) with a HR of 3.61 and a p of 1.5E−05 (Fig. 2C). On the other end of the spectra the first gene to reach significance was PDZ And LIM Domain 7 (PDLIM7) with a HR of 1.88 and a p of 7.2E−04 (Fig. 2D). Thus the variety of significant genes spanned a hazard rate ranging between 76% and 261% higher risk (when considering all HR values below one as inverted).

### 3.4 Genes associated with survival in basal breast cancer after adjuvant chemotherapy

Ten datasets had basal breast cancer patient samples with documented adjuvant chemotherapy, including GSE1456, GSE19615, GSE21653, GSE31519, GSE3494, GSE37946, GSE45255, GSE4611, GSE5327, and GSE69031. In the altogether 156 samples 1553 genes reached a FDR below 5%. When filtering for maximal expression over 1000 (n = 862) and cutoff over 100, 542 genes reached significance. The complete list of all genes related to relapse-free survival and ranked by the absolute HR values is provided in Supplemental Table 5. When examining the overrepresented biological processes among these genes, antigen processing and presentation (GO:0002504, p = 1.26E−06), T cell receptor signaling (GO:0050852, p = 7.09E−05), and immune response (GO:0006955, p = 1.62E−04) reached the highest significance. MHC class II receptor activity (GO:0032395, p = 2.5E−05) was the most significant molecular function. The seven biological process and three molecular function categories reaching significance in the GO analysis are listed in Supplemental Table 3.

### 3.5 Genes correlated to prognosis in untreated patients

The analysis was also executed by including only patients who did not receive a systemic treatment. Untreated estrogen-positive ERBB2 negative breast cancer patients (n = 686) were available from the GSE11121, GSE1456, GSE19615, GSE2034, GSE21653, GSE2990, GSE31519, GSE3494, GSE45255, GSE4922, GSE69031, and GSE7390 datasets, and the expression of all together 959 genes reached statistical significance in correlation to relapse-free survival (Supplemental Table 6). When comparing all genes related to survival in chemotherapy treated and untreated patients, 135 out of the combined 1515 genes were present in both lists (8.9%). Untreated basal breast cancer patients (n = 178) were accessible from the GSE11121, GSE19615, GSE2034, GSE21653, GSE2990, GSE31519, GSE3494, GSE45255, GSE69031, and GSE7390 datasets and 135 genes had a FDR below 0.05 in these (Supplemental Table 7). When compared to the genes related to chemotherapy response, 99.5% of genes were unique for one signature and only two genes (WARS and UBE2L6) were present in both lists.

### 3.6 Online survival analysis platform

The updated online analysis platform with transcriptomic and survival data of all 7830 breast cancer samples can be utilized at https://kmplot.com/analysis/index.php?p=service&cancer=breast. The correlation between survival and gene expression can also be evaluated for clinical cohorts not utilized in current project.

## 4 Discussion

Present study is based on multiple distinct steps. First, a sizeable database comprising thousands of breast cancer samples with clinical follow-up was assembled. The entire transcriptome was processed for each sample and redundant samples were removed. Then, survival analysis was made across all genes and the best performing genes were ranked for two cohorts with high clinical relevance: ER positive ERBB2 negative patients who received chemotherapy and basal breast tumors with chemotherapy.

Chemotherapy can improve the survival [20] but at the same time has significant risks due to the suppression of rapidly proliferating tissues including bone marrow (anemia, immunosuppression), hair follicles (alopecia), and the gastrointestinal tract (diarrhea) [21]. Chemotherapy can also have an effect on the central nervous system, lead to vomiting and early cognitive impairment [22]. Thus, it is crucial to select patients who get the most benefit from chemotherapy – different features are capable to assist in making this decision in different breast cancer subtypes.

ER positive ERBB2 negative tumors represent the largest cohort of breast cancer patients with over two third of all patients. The basic systemic therapy for these patients includes chemotherapy and endocrine therapy – we evaluated biomarkers of endocrine therapy previously [23]. The decision to administer chemotherapy can be based on clinical features including high stage or node positivity, or designation of high risk via gene expression profiles including Oncotype DX [24], [25] or EndoPredict [26]. Here, we run survival analysis for all genes in all chemotherapy treated ER positive ERBB2 negative patients to uncover genes correlated to survival following chemotherapy. With 692 significant genes we exposed a surprisingly large proportion of genes related to survival. Of note, when running the analysis using a less restricted criteria by including all patients who were not untreated, the number of significant genes was even higher. The significant prognostic biomarker genes achieved a hazard rate between 1.76 and 3.33 with a p value below 5.8e-04. When investigating common features of these genes by analyzing biological processes and molecular functions, GO categories related to cell division and chromatid segregation including microtubule binding were identified. These observations are in line with the previously described paradox correlation between chemosensitivity and low proliferation rates [27]. While the in depth discussion of individual genes is not in the scope of present study, a ranking of all significant genes in conjunction with the established threshold values help to quickly identify and filter genes related to chemotherapy response in this cohort in future genetic and transcriptomic studies.

Basal breast cancers that lack the expression of both steroid hormone receptors and ERBB2 represent approximately one sixth of all cases. Despite sensitivity to chemotherapy, these tumors have generally a poor prognosis [28]. In these tumors, the identification of good- and worse-prognosis cohorts has little value as even good-prognosis patients have a 20% risk of relapse [29]. Basal tumors are heterogenous and can be further subdivided into four molecular subtypes based on their transcriptomic fingerprint including the basal-like 1, basal-like 2, the mesenchymal, and the luminal androgen receptor subtypes [30]. While these subtypes have differences in clinical characteristics, the number of samples with clinical follow-up available in our database was not sufficient to perform a robust analysis across all genes within each subtype separately. When using all chemotherapy treated basal breast cancers we identified 246 genes significantly correlated to survival. The association with survival ranged between 1.88 and 3.61 for these genes. Only few gene ontology categories related to extracellular matrix organization and collagen catabolism were significantly overrepresented. Notably, then restricting the analysis to include only basal tumors with adjuvant chemotherapy, multiple gene ontology categories related to immune response reached statistical significance hinting on the involvement of the immune system. These observations correspond with the trend of advancing immune-mediated therapies in these patients [31].

Although the discussion of each gene associated with survival following chemotherapy is beyond the scope of present study, some interesting observations can be made when examining the list of significant genes. Well-known genes previously linked to chemotherapy response including TOP2A [32], MKI67 [33], ABC efflux pumps [34], or APOBEC3B [35] were related to survival following chemotherapy in ER positive tumors only, and none of these genes reached significance in basal tumors. The best performing genes associated with survival after chemotherapy in basal breast cancer were not significant in ER positive tumors. The reasons for these differences are most probably the different molecular characteristics related to the molecular subtypes. A recent study investigating clinical prognostic factors including TP53 status, grade, size, node positivity, ER and HER2 status, and age found that only nodal status was significantly associated with chemotherapy outcomes [36]. Combined, these results suggest that ultimately molecular and not clinical features will enable the prediction of response for chemotherapy in breast cancer.

While the two selected chemotherapy treated cohorts discussed above cover the largest chunk of breast tumors, there are sub-cohorts and other combination of clinical features. Our established online platform www.kmplot.com was extended with the entire updated database enabling the future validation and ranking of gene-expression based biomarkers in any sub-cohort of breast cancer. The analysis can help to identify biomarker candidates for subsequent *in vitro* validation studies [37], [38].

We have to mention a limitation of the presented approach. The collected and published clinical characteristics are incomplete for many of the available datasets. As a result, only a fraction of the total samples could be included in the statistical analyses. In addition, some detailed information, including the exact treatment protocol given was almost newer available.

In summary, by performing survival analysis across all genes we identified the best performing genes in chemotherapy treated estrogen-positive/ERBB2 receptor negative breast cancer and in basal breast cancer samples. A reference ranking for all significant genes is presented and the minimal hazard rates to reach clinically robust significance were established. The ranking and the established threshold values help to quickly identify and filter genes related to chemotherapy response in future genetic and transcriptomic studies.

## CRediT authorship contribution statement

**Balázs Győrffy:** Conceptualization, Data curation, Formal analysis, Funding acquisition, Investigation, Methodology, Project administration, Validation, Visualization, Writing - original draft, Writing - review & editing.

## Declaration of Competing Interest

The authors declare that they have no known competing financial interests or personal relationships that could have appeared to influence the work reported in this paper.
